# P-1738. Effectiveness of Clinical Pharmacist-Driven Antimicrobial Stewardship (ASP) in Bacteremic Patients in India

**DOI:** 10.1093/ofid/ofae631.1901

**Published:** 2025-01-29

**Authors:** Prasanna Kumar, Priscilla Rupali, Franklin Jose J, J Selvakumar

**Affiliations:** Christian Medical College, Velllore, Tamil Nadu, India; Christian Medical College, Vellore, Vellore, Tamil Nadu, India; Christian Medical College, Velllore, Tamil Nadu, India; Christian Medical College, Velllore, Tamil Nadu, India

## Abstract

**Background:**

Antimicrobial resistance is a prevalent problem, especially in low-middle-income countries. Infectious disease physicians are a scarce commodity and are unable to fully shoulder the complete responsibility of antimicrobial stewardship implementation in a health care setting. Thus, we set out to assess the utility of a Clinical pharmacist-led ASP in optimising antimicrobial use in bacteremic patients.

**Figure:**
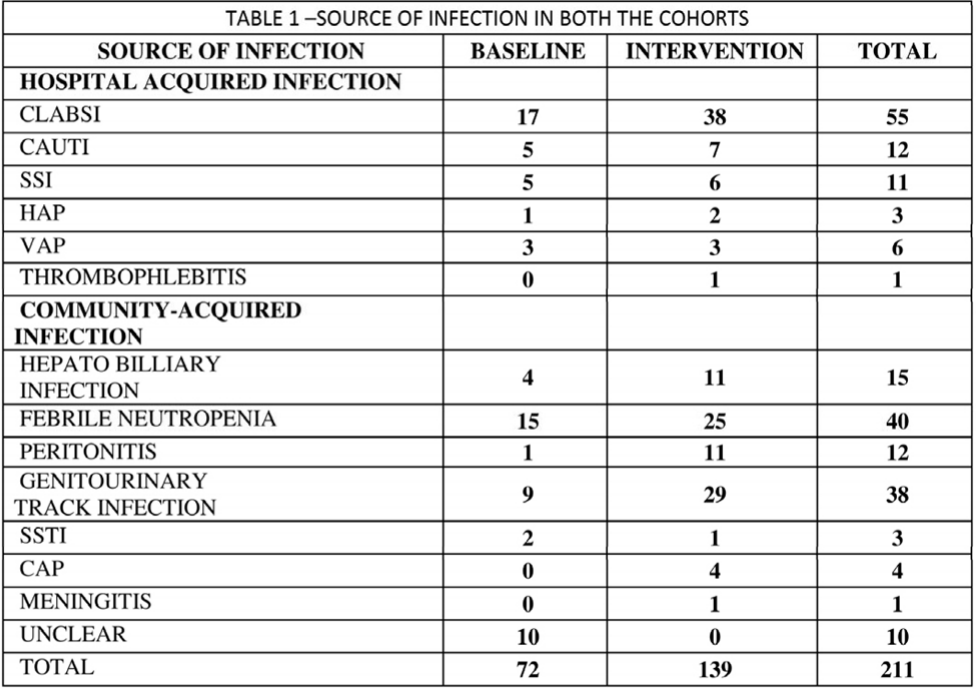

SOURCE OF INFECTION

**Methods:**

This is a prospective audit and feedback of bacteremic patients led by a clinical pharmacist with minimal input and sign-off from an ID specialist. A month of observation (July 2023) was followed by a 3-month intervention period from August to October 2023 in the medical and surgical speciality departments of a large tertiary care hospital in Southern India.

**Figure:**
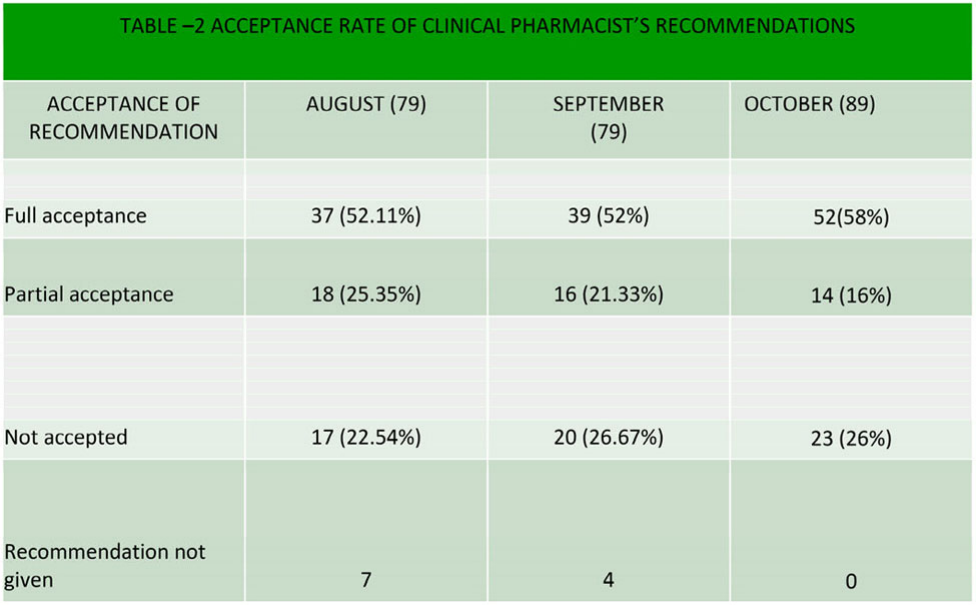

RECOMMENDATION ACCEPTANCE RATE

**Results:**

103 and 218 patients were noted to be bacteremic during baseline and intervention, respectively. The mean age was 48.2, and 66% were male. The majority of positive blood cultures were from Haematology (19%), Hepatology(15%), Nephrology(14%) and others (52%). The average length of stay was 19 days and 16.6 days in baseline and intervention phases, respectively. The predominant organisms were carbapenem-resistant and extended-spectrum beta-lactamase-producing enterbacteriaceae and coagulase-negative staphylococci (CoNS). The common sources of bacteremia were central line-associated bloodstream infection (CLABSI) and gut translocation during febrile neutropenia. (Table 1). The DOT in the observation phase was 1517/1000 Patient-Days (PD), and the intervention phase's average DOT was 1326/1000PD, and there was a significant reduction in antibiotic utilisation in the intervention (P=0.003). The length of therapy was 16.6 and 11.06 days, with in-hospital mortality of 13.6% and 8% in the observation and intervention phases, respectively. Clinical pharmacist recommendations were either fully or partially accepted in 75% of the cases in the intervention phase (Table 2).

**Conclusion:**

A clinical pharmacist-led ASP showed a reduction in significant antimicrobial use with 75% acceptance of recommendation and no difference in mortality even in bacteremic patients.

**Disclosures:**

**All Authors**: No reported disclosures

